# Understanding highand low-grade gliomas: VASARI criteria and MRI features

**DOI:** 10.1590/0100-3984.2024.57.e9

**Published:** 2024-11-18

**Authors:** Nina Ventura

**Affiliations:** 1 MD, PhD, Professor of Radiology at the Universidade Federal do Rio de Janeiro (UFRJ), Neuroradiologist at Fleury and at the Instituto Estadual do Cérebro Paulo Niemeyer, Rio de Janeiro, RJ, Brazil. Email: niventuraa@gmail.com

Adult gliomas constitute a diverse group of common primary tumors of the central nervous system^(1)^, previously classified by the World Health Organization (WHO) as low grade (WHO I and II) or high grade (WHO III and IV), on the basis of their histopathological, biological, and clinical characteristics^(2)^. The inclusion of isocitrate dehydrogenase (IDH) mutation status in the 2021 WHO classification marks a significant shift in the understanding and classification of gliomas^(3)^. Advances in molecular biology have revealed that genetic alterations, particularly IDH mutations, play a critical role in determining glioma behavior, prognosis, and response to treatment. This molecular insight has led to a more precise, biologically driven classification system^(3)^.

The presence or absence of IDH mutations is now recognized as one of the most important prognostic factors in glioma classification^(4)^. Low-grade gliomas (LGGs), such as astrocytomas and oligodendrogliomas, are mostly IDH-mutant and generally exhibit slower growth, longer progression-free survival, and better overall outcomes^(4,5)^. High-grade gliomas (HGGs), such as glioblastomas, are IDH wild-type and present as aggressive lesions with a poor prognosis^(4)^. Magnetic resonance imaging (MRI) plays a critical role in the diagnosis and characterization of gliomas and can provide important clues regarding tumor grade and aggressiveness^(6,7)^.

Imaging criteria such as the Visually AccesSAble Rembrandt Images (VASARI) feature set were developed to standardize the radiological analysis of these tumors, allowing the identification of characteristics that correlate with malignancy grade^(7,8)^. Among the VASARI features are contrast enhancement patterns, necrosis, hemorrhage, vasogenic edema, and the extent of the mass effect, which are most commonly observed in high-grade tumors. These imaging findings not only assist in preoperative tumor classification but also have prognostic implications^(7,8)^.

In a study recently published in Radiologia Brasileira, Campos et al.^(9)^ presented an analysis of 178 patients with gliomas and pathological confirmation, rated for tumor size, location, and tumor morphology using the VASARI feature set, developed in 2008. This method is potentially helpful when a brain biopsy cannot be performed or when there are questions about the pathological diagnosis. The authors found that hemorrhage, restricted diffusion, pial invasion, and enhancement were associated with HGGs, whereas absence of contrast enhancement was associated with LGGs. The article did not analyze advanced MRI sequences, mainly because they were not available for all patients or were not performed. Because these sequences are known to correlate well with glial neoplasm grade, future studies could include them in their analyses.

The differentiation of gliomas into HGGs and LGGs is critical for determining prognosis and treatment strategies^(3)^. MRI plays a pivotal role in this assessment, with certain imaging features, such as contrast enhancement, necrosis, and edema, offering essential insights into tumor grade^(6)^. The VASARI criteria provide a standardized method for evaluating these features, improving consistency in radiological assessment and facilitating communication among radiologists, oncologists, and neurosurgeons^(7,8,10)^.

Imaging technology continues to evolve, and future advancements, especially with artificial intelligence interfaces, deep learning, and the use of radiomics, may further enhance our ability to characterize gliomas noninvasively, leading to better-tailored therapies and improved outcomes for patients with these complex brain tumors^(11,12)^.
